# National survey on the current status of airway management in China

**DOI:** 10.1038/s41598-024-66526-8

**Published:** 2024-07-07

**Authors:** Yuewen He, Zhengze Zhang, Ruogen Li, Die Hu, Huan Gao, Yurui Liu, Hao Liu, Siqi Feng, Huihui Liu, Ming Zhong, Yuhui Li, Yong Wang, Wuhua Ma

**Affiliations:** 1 Guangzhou University of Chinese Medicine, Guangzhou, 510405 Guangdong People’s Republic of China; 2 Department of Anesthesiology, The First Affiliated Hospital of Guangzhou University of Chinese Medicine, 12 Jichang Road, Guangzhou, 510405 Guangdong People’s Republic of China; 3 Department of Anesthesiology, Fangcheng County People’s Hospital, Henan, People’s Republic of China

**Keywords:** Airway management, Intubation, Anesthesia, Survey, Respiratory signs and symptoms, Clinical trial design

## Abstract

Apparently, understanding airway management status may help to reduce risk and improve clinical practice. Given these facts, our team conducted a second survey on the current status of airway management for mainland China following our 2016 national airway survey. The national survey was conducted from November 7 to November 28, 2022. An electronic survey was sent to the New Youth Anesthesia Forum, where Chinese anesthesiologists completed the questionnaire via WeChat. A total of 3783 respondents completed the survey, with a response rate of 72.14%. So far, in 2022, 34.84% of anesthesiologists canceled or delayed surgery at least once due to difficult airway. For the anticipated difficult airway management, 66.11% of physicians would choose awake intubation under sedation and topical anesthesia, while the percentage seeking help has decreased compared to the 2016 survey. When encountering an emergency, 74.20% of respondents prefer to use the needle cricothyrotomy, albeit less than a quarter have actually performed it. Anesthesiologists with difficult airway training experience reached 72.96%, with a significant difference in participation between participants in Tier 3 hospitals and those in other levels of hospitals (*P* < 0.001). The videolaryngoscope, laryngeal mask, and flexible intubation scope were equipped at 97.18%, 95.96%, and 62.89%, respectively. Additionally, the percentage of brain damage or death caused by difficult airways was significantly decreased. The study may be the best reference for understanding the current status of airway management in China, revealing the current advancements and deficiencies. The future focus of airway management remains on training and education.

## Introduction

Effective airway management is one of the most important aspects of practicing clinical anesthesia, emergency, and intensive care, which is the cornerstone of maintaining patient life safety. Despite the gradual advances in current medical techniques, difficult airway remains challenging for anesthesiologists in clinical practice. Failed tracheal intubation and repeated intubation attempts are significantly associated with the occurrence of adverse events, including permanent brain damage and death^1^. The incidence of difficult airway for elective surgery in the operating room (OR) ranges from 0.5 to 8.5%^2^, with the situation being worse outside the OR^3^. It may be explained by the greater complexity of the patient’s status outside the OR and the urgency that may prevent a complete pre-intubation airway assessment^4^. Applying well-established algorithms for difficult airway may reduce the adverse effects of human factors and assist in appropriate clinical decision-making. As of 2019, more than 38 different difficult airway management algorithms have been published by various national or airway management societies^5^. Understanding anesthesiologists’ preferences for algorithms is necessary since the availability of practical cognitive tools can further improve airway management^6,7^. Moreover, the approach to airway management may vary depending on the hospital level or the clinical practice experience of anesthesiologists. Therefore, understanding the current status of airway management in medical institutions nationwide can improve clinical decision-making to a certain extent, especially in a country as vast as China, with varying levels of medical care and anesthesiologists’ training and experience^8^.

Our airway management team started a survey on the status of airway management in medical institutions in Guangdong Province, China, in 2011^9^. After that, a survey and analysis of the status of airway management in mainland China was initiated for the first time in 2016^10,11^. To gain insight into the changes and impacts associated with airway management over six years, we designed and conducted a new survey. It also compared with the 2016 survey to explore areas that have improved and remain inadequate. Collecting and analyzing factual information from clinical practice helps to reduce the risks associated with difficult airway and enhance clinical decision-making.

## Methods

This national survey began on November 7, 2022, and closed on November 28. The valid period for the survey started from November 2016 to November 2022, and the data within this six-year interval were considered to be included in the study. The target respondents were anesthesiologists in mainland China. This survey was approved and endorsed by the First Affiliated Hospital of Guangzhou University of Chinese Medicine (Approval Number: No. K-2022-103).

### Survey population

This national survey of airway management was completed at the New Youth Anesthesia Forum (http://www.xqnmz.com), the largest anesthesia platform with over 80,000 registered anesthesiologists in China. We distributed the questionnaire to all anesthesiologist members via WeChat (Tencent Holdings Limited, Shenzhen, China). Respondents could use a computer or mobile terminal to complete the questionnaire. In addition, we limit each IP address (Internet Protocol Address, IP) to be filled in only once to ensure the most realistic representation. The availability of airway devices and adverse events were filled out only by the director of the Department of Anesthesiology (head of the department) to avoid duplication. Sources of the information reported by participants for the 2022 survey were based on records, databases, or memories of events between 2016 and 2022.

### Survey design

The questionnaire design was derived from the summaries of our airway management team in the Guangdong province airway management survey in 2011^9^ and the first Chinese airway management survey in 2016^10^. Since the questionnaire is intended for the community of Chinese anesthesiologists, the language of our questionnaire is Chinese. The calibration and translation of relevant standardized terminology are based on the latest ASA (American Society of Anesthesiologists) guideline^12^, DAS (Difficult Airway Society) guideline^13^, and CSA (Chinese Society of Anesthesiologists) guideline^14^. After review by the team members and experts, the questionnaire was uploaded to the New Youth Anesthesia Forum and distributed by WeChat.

### Survey program

The survey covers 31 provinces, municipalities, and autonomous regions in mainland China, making it the most extensive survey on airway management in China, with the largest number of participants. The questionnaire was designed with 54 questions in nine categories (Supplemental Table S1). The first seven categories of questions (1–39) were open to all survey respondents, while the eighth to ninth categories of questions (40–54) could be answered only by the director of the Department of Anesthesiology.

The survey was organized according to the following categories: essential characteristics of the respondent; how to assess the airway; anticipated difficult airway management; unanticipated difficult airway management; the application of FONA techniques; airway management outside the operating room; training and learning in airway management; availability of noninvasive airway devices and emergency airway devices within the Department of Anesthesiology; and the occurrence of airway-related adverse events from 2016 to 2022. To minimize bias in the questions answered, most of the questions used a “yes” or “no” format or selected the best answer from two to five alternatives. Results were presented as the percentage of respondents for each question. No monetary compensation was provided for any form of participation in this survey.

### Statistical analysis

All backend data can be exported from the Wenjuanxing platform (https://www.wjx.cn) and analyzed with R software (version 4.2.1). Statistical analyses were performed using R software (version 4.2.1) with the χ^2^ test or Fisher’s exact test. All tests were two-tailed with a type I error rate of 0.05. In this study, we considered *P* < 0.05 to be statistically significant.

### Ethics approval and consent to participate

This survey was approved and endorsed by the First Affiliated Hospital of Guangzhou University of Chinese Medicine (Approval Number: No. K-2022–103). All methods were carried out in accordance with relevant guidelines and regulations. Informed consent was obtained from all subjects.

## Results

**Figure 1:**
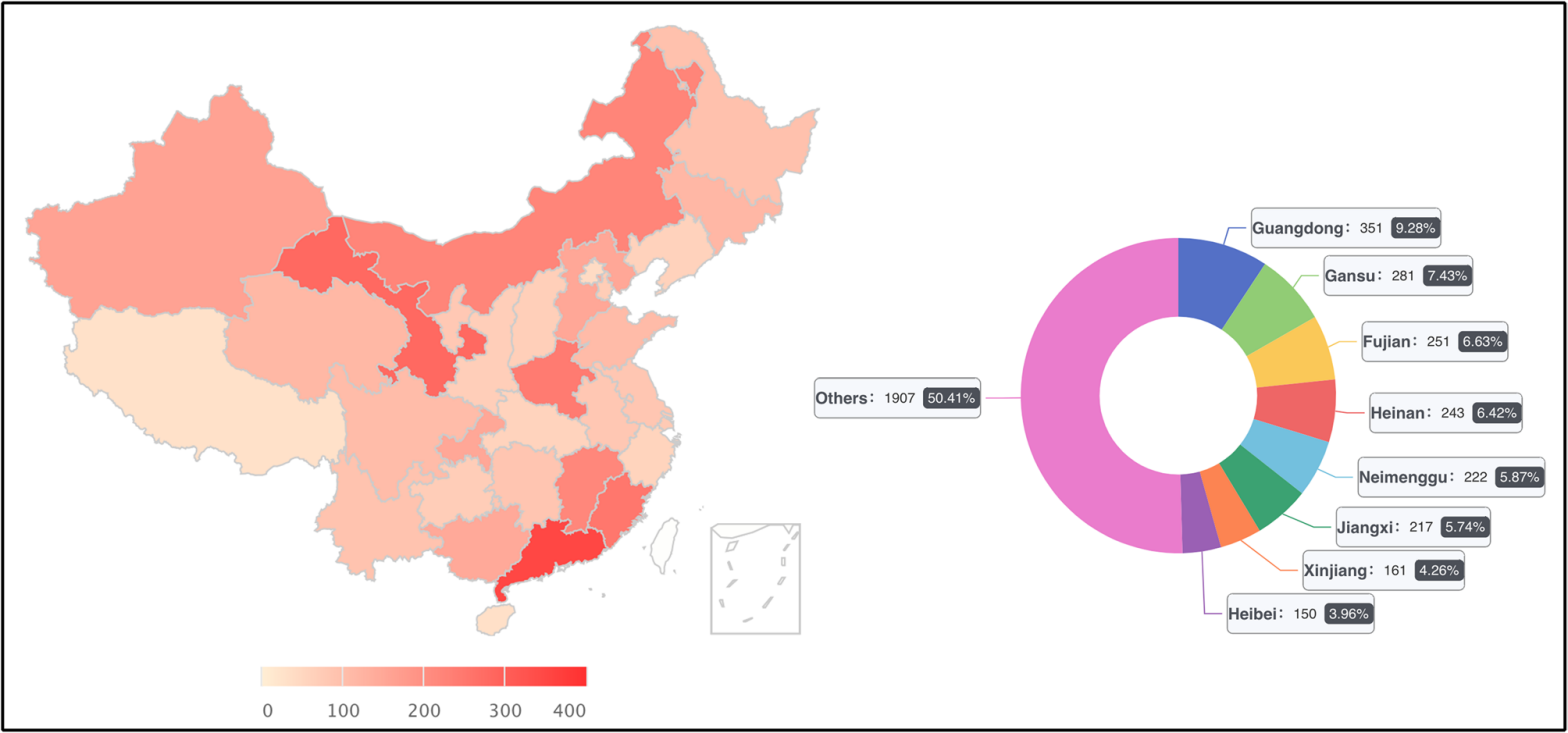
Regional distribution of respondents.

The survey covered anesthesiologists in 31 provinces, municipalities, and autonomous regions in mainland China, with 5244 readers and 3783 respondents, a response rate of 72.14%, according to the backend server (Fig. 1; Table 1). Two investigators collected responses independently and manually checked to ensure that only one response from each anesthesiology department was counted.

**Table 1:** Basic characteristics of respondents.

	Numbers (%)
Years of work experience	
Less than 5 years	383(10.12%)
5–10 years	683(18.05%)
More than 10 years	2717(71.82%)
Tiers of hospitals	
Tier 3	2546(67.30%)
Tier 2	1157(30.58%)
Others	80(2.11%)
The director of the anesthesiology department	1385(36.61%)
In the past 6 years, have you ever canceled or delayed surgery due to difficult airway: Yes	1318(34.84%)
In the past 6 years, has your department encountered the use of FONA technique to rescue patients: Yes	1161(30.69%)

### Background information for participants

Figure 1 displays the characteristics of the regional distribution of respondents. The darker the map’s color, the higher the amount of access from that region. Also, the donut map showed geographical characteristics (Fig. 1). Through the collection and analysis of respondents’ background information, there were 2546 anesthesiologists from Tier 3 hospitals, accounting for 67.30%, and 1237 anesthesiologists from Tier 2 hospitals and other hospitals, accounting for 32.70%. Hospitals in China are divided into three levels according to their ability to provide medical services, medical education, and scientific research. Tier 3 hospitals are China’s top hospitals, usually general hospitals in cities with more than 500 beds that provide the best medical care^15^. Tier 1 hospitals, on the other hand, are usually rural or community hospitals that provide only the most basic medical care. A total of 2717 anesthesiologists with more than ten years of working experience participated in the survey, representing 71.82%. Additionally, the director of the anesthesiology department accounted for 36.61% (Table 1).

### Evaluation of difficult airway

**Figure 2:**
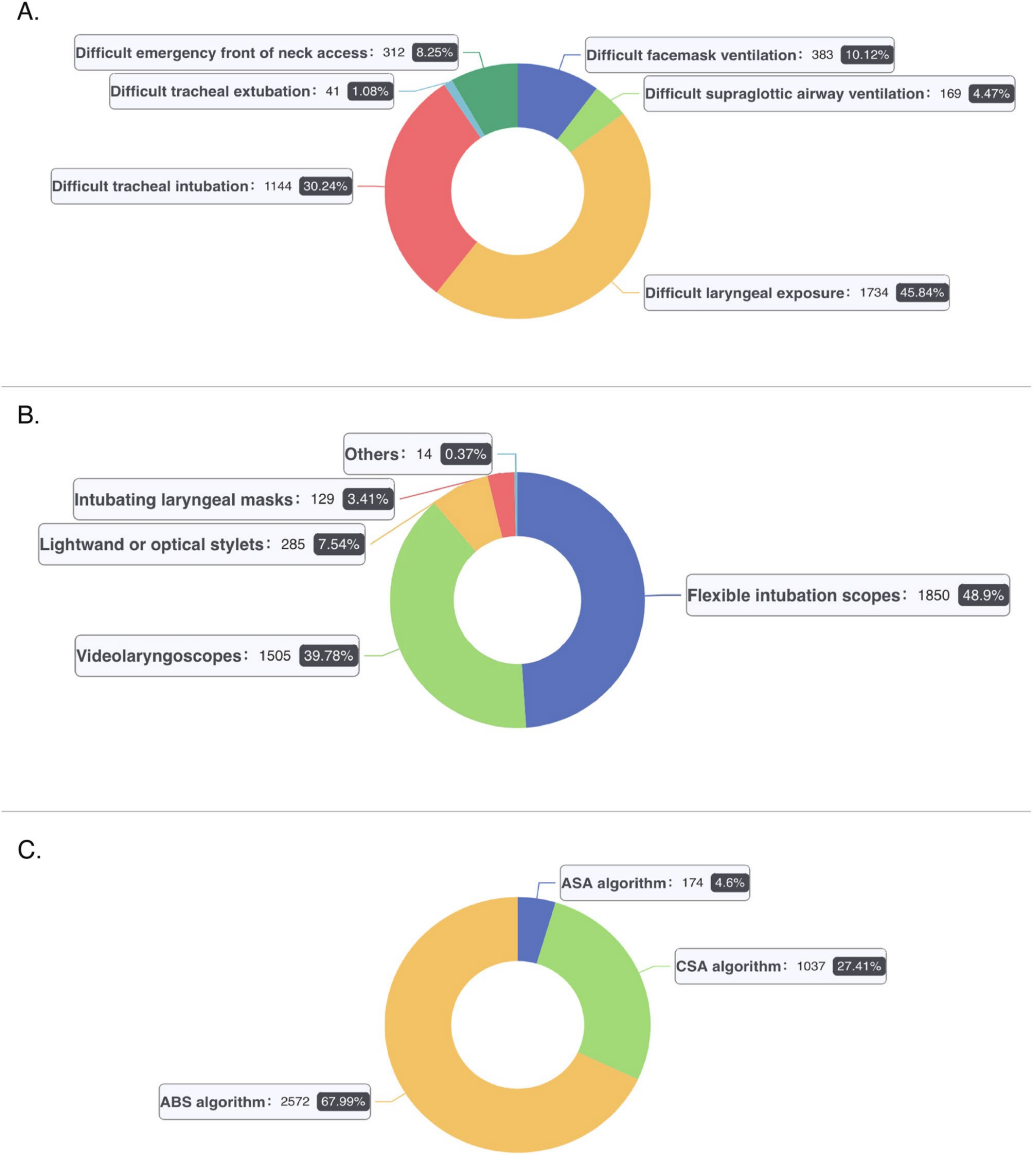
Pie chart of the most common types of DA (**A**); Pie chart of the preferred intubation device for respondents when encountering anticipated DA (**B**); Pie chart of the difficult airway management algorithm that respondents indicated they were most familiar with (**C**).

So far, in 2022, 1318 (34.84%) anesthesiologists reported experiences of delayed or stopped surgeries due to difficult airway. The most common type of difficult airway was difficult laryngeal exposure (DLE), accounting for 45.84%, followed by difficult intubation (30.24%), difficult mask ventilation (10.12%), difficult emergency front of neck access (8.25%), difficult supraglottic airway (4.47%), and difficult extubation (1.08%) (Fig. 2A). In addition, the top three airway assessments reported by respondents were mouth opening 95.40%, thyromental distance 86.44%, and atlanto-occipital joint movement 69.13% (Supplementary Table S1; Fig. S1).

### Anticipated difficult airway management

Participants indicated that, in general, their preferred devices for managing difficult airways were video flexible intubation scopes (48.90%), videolaryngoscopes (39.78%), lightwand or optical stylets (7.53%), intubating laryngeal masks (3.41%) and others (0.37%) (Fig. 2B). For anticipated difficult airway management, 66.11% of anesthesiologists would prefer awake intubation under sedation and topical anesthesia, and 22.31% would choose intubation under general anesthesia with preserved spontaneous breathing. However, 11.58% of anesthesiologists would still choose rapid sequence anesthesia induction (Supplementary Table S1; Table 2). A total of 68.17% of respondents expressed that they routinely seek help from superiors or colleagues (Table 2). The majority of respondents refrained from choosing awake intubation under sedation and topical anesthesia owing to concerns about patient non-acceptance (38.81%) or the patient expressed refusal (27.23%). When performing awake intubation under topical anesthesia, only 67.71% of anesthesiologists stated routine use of anticholinergics to reduce oropharyngeal secretions. Supplementary Table S2 shows significant differences in the selection of intubation devices and preferred management approaches among anesthesiologists between Tier 3 hospitals and other hospitals.

**Table 2:** Comparison of airway management survey results in mainland China in 2016 and 2022.

	Survey in 2016 (N = 1935)	Survey in 2022 (N = 3783)	*P*-value
Tiers of hospitals			
Tier 3	962(49.71%)	2546(67.30%)	< 0.001
Others	973(40.29%)	1237(32.70%)	< 0.001
The most common approaches to predicting difficult intubation (Top three)	Mouth opening (86.87%) Mallampti test (55.34%) Thyromental distance (39.27%)	Mouth opening (95.40%) Thyromental distance (86.44%) Atlanto − occipital joint movement (69.13%)	NA
Have you experienced canceling or delaying surgery due to difficult airway in recent years: Yes	1224(63.24%)	1318(34.84%)	< 0.001
Anticipated difficult airway management			
AIST	1314(67.90%)	2501(66.11%)	0.182
GASB	295(15.25%)	844(22.31%)	< 0.001
RSAI	326(16.85%)	438(11.58%)	< 0.001
Reasons for not choosing awake intubation			
Worried that patients will not accept	NA	1468(38.81%)	NA
Unfamiliarity	1024(52.94%)	647(17.10%)	< 0.001
Long operation time	358(18.49%)	350(9.25%)	< 0.001
High failure rates	195(10.08%)	288(7.61%)	0.002
Patient rejection	358(18.49%)	1030(27.23%)	< 0.001
Are anticholinergic drugs routinely used for awake intubation: Yes	NA	2541(67.17%)	NA
Will you ask for help if you encounter anticipated difficult airway			
Routinely	1665(86.04%)	2579(68.17%)	< 0.001
Occasionally	256(13.24%)	962(25.43%)	< 0.001
Handling independently	14(0.72%)	242(6.40%)	< 0.001
What’s the biggest concern about intubation outside the OR			
Difficult airway	1351(69.84%)	2384(63.02%)	< 0.001
Full stomach	432(22.30%)	813(21.49%)	0.490
Surrounding environmental impact	147(7.60%)	172(4.55%)	< 0.001
Hemorrhage from the respiratory tract	NA	405(10.71%)	NA
Others	5(0.26%)	9(0.24%)	0.902
How to confirm the position of the ETT when intubating outside the OR			
Auscultation	1587 (82.02%)	2865(75.73%)	< 0.001
Chest rise	145(7.48%)	340(8.99%)	0.062
Capnography	113(5.86%)	397(10.49%)	< 0.001
Flexible intubation scope	66(3.42%)	162(4.28%)	0.128
Others	24(1.22%)	19(0.50%)	0.003
Have you ever performed below FONA technique			
Needle cricothyrotomy	278(14.37%)	904(23.90%)	< 0.001
Surgical cricothyrotomy	88(4.55%)	296(7.82%)	< 0.001
Tracheotomy	68(3.51%)	300(7.93%)	< 0.001
Have you ever attended difficult airway training: Yes	983(50.85%)	2760(72.96%)	< 0.001
What type of devices would you most like to be trained on			
Laryngeal mask	46(2.38%)	55(1.45%)	0.016
Videolaryngoscope	105(5.43%)	271(7.16%)	0.142
Flexible intubation scope	509(26.30%)	1241(32.80%)	< 0.001
Lightwand	58(3.00%)	137(3.62%)	0.249
Emergency airway devices	1217(62.89%)	2079(54.96%)	< 0.001
Have you ever received the below training			
Retrograde intubation	397(20.52%)	1124(29.71%)	< 0.001
Needle cricothyrotomy	578(29.87%)	1964(51.92%)	< 0.001
Surgical cricothyrotomy	326(16.85%)	1049(27.73%)	< 0.001
Tracheotomy	243(12.56%)	831(21.97%)	< 0.001
Jet ventilation	NA	1053(27.84%)	NA
Does your department have the below devices*			
Videolaryngoscope	1410(72.91%)	1346(97.18%)	< 0.001
Lightwand or optical stylet	1005(51.96%)	834(60.22%)	< 0.001
Flexible intubation scope	771(39.87%)	871(62.89%)	< 0.001
Bougie or airway exchange catheter	445(23.00%)	472(34.08%)	< 0.001
Laryngeal mask	1818(93.89%)	1329(95.96%)	0.131
Video laryngeal mask	NA	195(14.08%)	NA
Video endotracheal tube	NA	166(11.99%)	NA
Jet ventilation device	336(17.37%)	200(14.44%)	0.027
Needle cricothyrotomy kit	538(27.82%)	501(36.17%)	< 0.001
Difficult airway management emergency kit or cart	659(34.07%)	676(48.81%)	< 0.001
Has your department identified any of the below issues due to difficult airway			
Cardiac arrest without sequelae	NA	88(6.35%)	NA
Brain damage or dead event	267(13.79%)	71(5.13%)	< 0.001
None of the above occurred	1668(86.21%)	1195(86.28%)	0.989
Others	NA	31(2.24%)	NA

*The following questions of the 2022 survey will be answered by the director of the Department of Anesthesiology only (N = 1385).

*AIST* Awake intubation under sedation and topical anesthesia, *GASB* General anesthesia with preserved spontaneous breathing, *RSAI* rapid sequence anesthesia induction, *OR* Operation room, *ETT* Endotracheal tube, *FONA* Front of neck access.

### Unanticipated difficult airway management

When encountering a situation where the glottis cannot be exposed after administration of muscle relaxant, approximately 63.31% of anesthesiologists would choose to blindly insert once while ensuring ventilation and waiting for help from superiors (Supplementary Table S1). If the first attempt failed, 47.71% of respondents would switch to another device for intubation, 43.22% would seek help, and 8.33% would keep ventilated and allow patients to wake up before deciding. After failed intubation, 73.20% of respondents preferred laryngeal masks to maintain oxygenation, besides regular masks, followed by oropharyngeal airways and Combitube (Supplementary Table S1).

### Front of neck access emergency techniques

**Figure 3:**
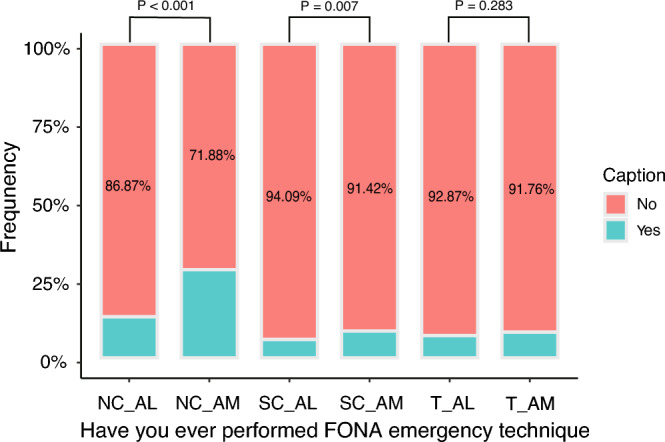
Comparison of anesthesiologists with more than ten years of working length and anesthesiologists with less than ten years of working length (AL) in terms of emergency FONA techniques. *NC* Needle cricothyrotomy, *SC* Surgical cricothyrotomy, *T* Tracheotomy.

There were 1,161 anesthesiologists (30.69%) who reported that they had encountered using the front of neck access (FONA) emergency techniques to save patients in the past six years (Supplementary Table S1). In total, 2807 respondents (74.20%) who reported encountering cannot intubate cannot ventilate (CICV) situations would prioritize needle cricothyrotomy to establish FONA, although only 904 respondents (23.90%) had actual experience with the procedure. Of the 3783 participants, only 296 anesthesiologists (7.82%) had performed a surgical cricothyrotomy, and 300 anesthesiologists (7.93%) had conducted a tracheotomy. Indeed, in most Chinese hospitals, tracheotomies are performed by non-anesthesiologists, as reported by 80.68% of the respondents (Table 2). Furthermore, there were significant differences between anesthesiologists with more than ten years of working length and anesthesiologists with less than ten years of working length regarding needle cricothyrotomy (*P* < 0.001) and surgical cricothyrotomy (*P* = 0.007) (Fig. 3; Supplementary Table S3).

### Difficult airway management outside the operating room

According to Table 2, anesthesiologists considered difficult airway (63.02%) to be the most challenging situation during intubation outside the OR. Outside the OR, the three most critical intubation devices that anesthesiologists believed they required to carry were videolaryngoscopes (92.55%), manual resuscitators (81.79%), and laryngeal masks (71.87%), with 12% proportion of video flexible intubation scopes (Table 2). In addition, 75.53% of respondents chose auscultation to determine endotracheal tube (ETT) location, 10.49% for capnography, and only 4.28% used video flexible intubation scope (Table 2).

### Difficult airway management training

**Figure 4:**
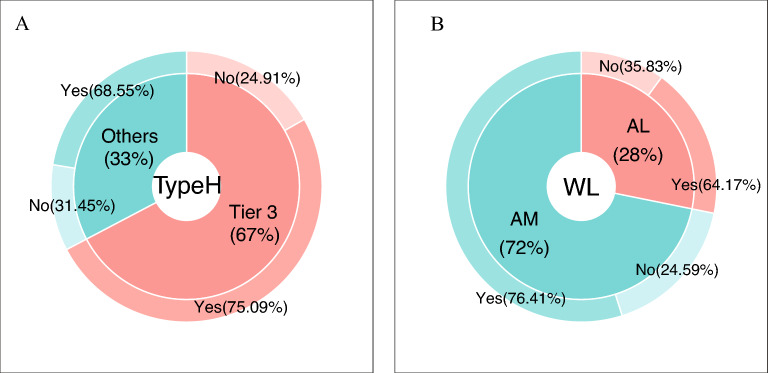
Proportion of anesthesiologists with or without airway training in tertiary hospitals versus other hospitals (**A**); Proportion of anesthesiologists with more than ten years of experience (AM) versus anesthesiologists with less than ten years (AL) who have or have not received airway training (**B**). *TypeH* Type of hospital, *WL* Working length.

Of the respondents, 72.96% indicated they had participated in difficult airway training. Figure 4 demonstrated a significant difference in training involvement between anesthesiologists serving in Tier 3 hospitals compared to those working at other hospitals (*P* < 0.001) (Fig. 4A), as well as between anesthesiologists with different years of working length (*P* < 0.001) (Fig. 4B). More than half of the participants (54.96%) expressed their willingness and expectation for training in emergency airway techniques. Table 2 displays the percentage of respondents who have received training in emergency airway techniques for retrograde intubation (29.71%), needle cricothyrotomy (51.92%), surgical cricothyrotomy (27.73%), tracheotomy (21.97%), and jet ventilation (27.84%). Additionally, 67.99% of respondents identified the ABS algorithm (Supplementary Table S1) as their most familiar difficult airway management algorithm, followed by the CSA algorithm (27.41%) and the ASA algorithm (4.60%), respectively (Fig. 2C).

### Availability of airway devices and emergency airway devices

Table 2 compares the availability of noninvasive ventilation devices and emergency airway devices in the 2016 and 2022 national airway surveys. According to the 2022 survey, 97.18% and 95.96% of the anesthesiology departments were equipped with videolaryngoscopes and laryngeal masks. The availability of other noninvasive airway equipment or emergency devices also increased substantially (Table 2).

### Adverse events related to difficult airway

As stated by the director of the Department of Anesthesiology, the incidence of airway adverse events from 2016 to 2022 was 11.48%, of which 6.35% were cardiac arrests without sequelae and 5.13% were brain damage or death (Table 2).

## Discussion

It is the national survey of the current status of difficult airway management in China, covering hospitals at all levels in 31 provinces, municipalities, and autonomous regions in mainland China. According to Candlish^16^ and colleagues, the minimum number of valid participants for the survey should equal ten times the number of questions. The survey set 54 questions with 3,873 respondents (71.7 times), which means the survey results may be highly credible. A considerable upsurge could be observed in the anesthesiologists with difficult airway training experience (72.96% vs. 50.85%), as well as the availability of videolaryngoscopes (97.18% vs. 72.91%) and video flexible intubation scopes (62.89% vs. 39.87%) compared to the 2016 survey. When confronted with CICV situations, 74.20% of the respondents preferred needle cricothyrotomy to surgical cricothyrotomy. Meanwhile, airway adverse event rates were significantly reduced. Yet intractable airway problems persist. Difficult airway management remains one of the core tasks of anesthesia safety. To help lower risks and improve clinical practice, the current survey may be the finest reference for understanding airway management status in China.

Mouth opening, thyromental distance, and atlanto-occipital joint movement are the most commonly used approaches in airway evaluation. Even though some studies suggest the rapid bedside airway evaluation test seems to have limited sensitivity or specificity and cannot be effectively used for all patients^17^, it is nonetheless widely used in China. The number of anesthesiologists per 100,000 population in China (5.12 per 100,000) lags far behind the level of high-income countries (17.96 per 100,000) and is even lower than some developing countries^18^. Additionally, the workload rise related to anesthesia is far higher than the growth in the number of anesthesiologists^15^. Despite the hectic schedule of Chinese anesthesiologists, there is a need to enhance preoperative comprehensive interviews and assessments of patients to ensure perioperative airway safety. It is the section that we need to strengthen.

For the anticipated difficult airway, several leading airway guidelines and algorithms recommend the application of preserving spontaneous breathing techniques^6,12,14,19,20^. However, 11.58% of participants still opted for rapid sequence anesthesia induction. Being a relative contraindication for anticipated difficult airway and a known risk factor for failed intubation, which should not be used at this point^21^. So, why not follow the difficult airway guidelines and algorithms? As stated by the respondents, concern about patient non-acceptance and lack of expertise in awake intubation techniques were the main reasons. Indeed, awake intubation with a video flexible intubation scope is sophisticated and calls for persistent practice. Concerns by the anesthesiologist may indicate a lack of confidence in themselves. Acknowledging this, additional training is necessary to strengthen the anesthesiologists’ confidence in performing the procedure.

Preoperative airway evaluation approaches may be practical, yet not foolproof. Even while there is a slim probability that an unanticipated difficult airway will be encountered during the perioperative period, the severe complications that could result from it could be fatal^1^. When difficult laryngeal exposure occurred after applying muscle relaxants, only 43.22% of participants chose to seek help after one blindly failed attempt. Similarly, merely 68.17% of participants would seek help for an unanticipated difficult airway, a dramatic drop from 86.04% six years earlier. The significant decrease in help-seeking for airway issues do not match management guidelines and algorithms, possibly due to the development and availability of a wide range of intubation devices^22^. Indeed, China has made great strides in equipping airway devices. Similar to the UK survey results^23^, the availability of noninvasive airway devices was overall significantly higher than it was in 2016, particularly concerning videolaryngoscopes (97.18%), laryngeal masks (95.96%) and flexible intubation scopes (62.89%). Availability of difficult airway management kits or carts has also increased significantly (48.81%), though it remains far below the 70–90% level in developed countries^24^. Using airway emergency kits or carts is advisable, for providing better ability to manage anticipated and unanticipated airway events^25^. The Department of Anesthesiology or the OR should be equipped with emergency airway devices whenever possible, as it is directly related to reducing the incidence of adverse airway events^26^. Although airway devices can address airway problems to some extent, we need to emphasize that seeking help and limiting intubation attempts are the primary principles in any circumstance where an airway issue arises.

When intubation fails after induction and cannot sustain oxygenation, the patient will progress to CICV. At this point, establishing FONA is an efficient strategy ^27^. ASA and DAS guidelines recommended surgical cricothyrotomy as a means of resuscitation. The national survey revealed that only 296 respondents (7.82%) had performed this procedure. While 2807 respondents (74.20%) encountering CICV would prefer needle cricothyrotomy. As needle cricothyrotomy is less invasive, rapid, and relatively simple, it is more appealing to Chinese anesthesiologists^28^. However, Hubble^29^ and colleagues observed that patients who underwent surgical cricothyrotomy (90.5%) had a higher success rate than needle cricothyrotomy (68.5%). Even if the quality of the evidence may not have been high, it nevertheless suggests that it is insufficient for anesthesiologists to be equipped with a single FONA technique ^30^. Multivariate regression demonstrated that Anesthesiologists with more than ten years of working length had significantly more incredible experience than anesthesiologists with less than ten years of working length in performing needle cricothyrotomy (*P* < 0.001) and surgical cricothyrotomy (*P* = 0.007). At the same time, there was no difference between the two groups regarding tracheotomy (*P* = 0.283). This is because tracheotomies are typically carried out by non-anesthesiologists in China, as confirmed by the majority of respondents (80.68%).

Human-related factors account for 40% of adverse outcomes in managing CICV, underscoring the importance of human factors in airway management^31^. The potential negative impacts of human factors may be amplified further owing to the emergency nature of difficult airway and the complexity of airway management algorithms. The amount of information that needs to be processed during a crisis frequently exceeds our cognitive ability. As a consequence of cognitive overload, the anesthesiologist alone may make poor decisions or “lose sight of the big picture” in favor of the immediate goal of intubation^32^. The correct way to handle difficult airway is to seek help and collaborate to bridge the knowledge gap since managing difficult airway independently in an emergency is tricky for anesthesiologists who lack experience. Thus, it is essential to keep reinforcing the notion that seeking help is an effective method to mitigate human factors’ impact.

Intubation outside the OR is a critical test of the anesthesiologists’ ability to maintain the airway and frequently represents emergencies, device limits, or inadequate preparation^33^. As a result, the risk of intubation failure and complications is higher with difficult airway outside the OR^34^. The survey revealed that, at 63.20%, slightly lower than the previous survey’s finding of 69.84%, difficult airway remained the most problematic event for anesthesiologists performing intubations outside the OR. So perhaps the survey of emergency intubations outside the OR best captures the genuine mindset of anesthesiologists toward worries about challenging airway issues. Also, after intubation outside the OR, it is crucial to promptly confirm the proper location of the ETT in patients with difficult airway. Up to 75.73% of respondents still rely on auscultation to confirm ETT location. The adoption of capnography was only 10.49%, albeit an increase compared to the 2016 survey (5.86%; *P* < 0.001). Although auscultation is easy to apply, it is inaccurate in determining the location of the ETT. As early as 2005, the American Heart Association had already recommended capnography to determine ETT location^35^. The survey discovered that anesthesiologists tended to use simple methods or prior experience to confirm the location of ETT, which is contrary to current guidelines and is a quite risky approach^36^. We emphasize that the capnography waveform is the gold standard for confirming ETT placement and proper ventilation for any airway management protocol. Consequently, as an aspect of quality improvement in airway management, additional training and learning programs should be provided so that using portable capnography outside the OR in China as a trustworthy standard for verifying the placement of ETT.

How can anesthesiologists get ready for difficult airway in a crisis? Training in airway devices, emergency FONA techniques, and airway management algorithms are crucial components^37^. According to the study, anesthesiologists working at Tier 3 facilities received a significantly higher percentage of airway management training. In mainland China, the quality of medical care varies. In fact, the vast majority of university hospitals in China are Tier 3 hospitals. Affiliation between Tier 3 hospitals and medical universities is usually joint, reflecting the synergy between clinical care and medical education. These hospitals tend to have a higher concentration of well-educated, highly qualified anesthesiologists. Meanwhile, there is a snowball effect of these hospitals being able to provide a platform for more academic mentoring or hands-on training opportunities. Thus, the training and education of anesthesiologists in lower-level hospitals should be focused on strengthening. Emergency airway devices (54.96%) and flexible intubation scopes (32.80%) ranked as the top 2 most desired devices to be trained by the respondents, reflecting the urgent need for anesthesiologists to master complex airway management techniques. Regarding training, the apprenticeship approach, which relies on hands-on learning in a clinical setting, has been used in traditional medical education for more than a century^38^. Until now, opportunities for novice anesthesiologists to acquire intricate and challenging airway techniques are dwindling as novel and efficient intubation devices become accessible. However, novel devices are frequently utilized regularly without being formally taught^39^. The fact that many anesthesiologists learn their skills through self-working experience, which can have adverse consequences to patients. Consequently, it is recommended that all airway management workshops or difficult airway training programs should enhance training in airway devices, especially complex emergency airway techniques.

Most anesthesiologists endorse difficult airway management algorithms and guidelines that emphasize fundamental principles and set uniform criteria for various settings. More than 30 difficult airway management algorithms are used globally because a single algorithm cannot capture the complexity of difficult airway issues^5^. Therefore, knowing the anesthesiologist’s preference for the algorithm is necessary. The survey found that 44.12% of anesthesiologists claim that the ASA algorithm was complicated and hard to recall. In contrast, only 4.60% of the respondents were familiar with the ASA algorithm, with similar findings from a European airway management survey^40^. An “overly informative” algorithm can cause confusion when confronting emergency airway issues and hinder proper and efficient application^5^. Of the three representative difficult airway management algorithms, 67.99% of anesthesiologists stated that the ABS algorithm was the most familiar, given its practical, safe, and simple features. Consequently, practical and simple-to-remember algorithms may be our future endeavor, whichwill be welcomed and adopted by anesthesiologists. In summary, airway techniques and guidelines training should be carried out throughout our careers to enhance our airway management expertise^41^. It is worth noting that training and education rely mostly on systematic and routine arrangements in the department. However, the survey indicated that nearly 20% of the anesthesiology departments had never scheduled difficult airway training and almost half of the departments lack a quarterly practice of sharing difficult airway cases. All of these reflect how the focus on difficult airways is still far from sufficient and has to be strengthened.

Indeed, some limitations exist in our survey. Initially, our findings do not represent the degree of experience or perception of all Chinese anesthesiologists due to sampling size restrictions. Moreover, the survey respondents provided their self-reported data. The respondents’ subjective data can introduce some bias to the findings. Furthermore, the survey respondents provided self-reported data, possibly introducing bias to the findings. The valid period for this survey is 2016 to 2022, and it is not assured whether all data or events reported during this period were documented or simply relied on memory recall, which may create information bias. Also, due to the limited number of questions, it was hard to cover every facet of the difficult airway in the questionnaires completely. Finally, the accuracy of the findings could not be independently verified due to the survey study’s limitations, and future pertinent randomized controlled trials are required to investigate this further.

## Conclusions

Several accomplishments were revealed in the second national survey on the status of airway management. While tricky airway issues persist. Hence, we are alerted again that difficult airway management remains one of the challenging tasks facing anesthesiologists. In light of these findings, we need to consider how we may optimize airway management, particularly at primary hospitals with limited medical resources and among junior anesthesiologist groups. Anesthesiologists need to give airway management enough consideration and put forth the effort to follow guidelines or algorithms. Meanwhile, it’s crucial to emphasize the importance of seeking help when managing airway issues. Last but not least, training should be implemented into the daily work of anesthesiologists, especially for sophisticated emergency airway techniques.

### Supplementary Information

Supplementary Information.

## Data Availability

All data generated or analyzed during this study are included in this published article and its supplementary information files.

## Abbreviations

difficult airway: Difficult airway
OR: Operating room
ASA: American Society of Anesthesiologists
DAS: Difficult Airway Society
CICV: Cannot intubate
AM: Anesthesiologists with more than ten years of working length
AL: Anesthesiologists with less than ten years of working length
FONA: Front of neck access
ETT: Endotracheal tube
UK: United Kingdom

## References

[1] Cook TM, Woodall N, Frerk C (2011). Major complications of airway management in the UK: Results of the fourth national audit project of the royal college of anaesthetists and the difficult airway society. Part 1 anaesthesia. Br. J. Anaesth..

[2] Nørskov AK, Rosenstock CV, Wetterslev J, Astrup G, Afshari A, Lundstrøm LH (2015). Diagnostic accuracy of anaesthesiologists’ prediction of difficult airway management in daily clinical practice: A cohort study of 188 064 patients registered in the Danish Anaesthesia Database. Anaesthesia.

[3] Cook TM, Woodall N, Harper J, Benger J (2011). Major complications of airway management in the UK: Results of the fourth national audit project of the royal college of anaesthetists and the difficult airway society. Part 2 intensive care and emergency departments. Br. J. Anaesth..

[4] Scott JA, Heard SO, Zayaruzny M, Walz JM (2020). Airway management in critical illness: An update. Chest.

[5] Edelman DA, Perkins EJ, Brewster DJ (2019). Difficult airway management algorithms: A directed review. Anaesthesia.

[6] Gómez-Ríos M, Sastre JA, Onrubia-Fuertes X, López T, Abad-Gurumeta A, Casans-Frances R, et al. Spanish society of anesthesiology, reanimation and pain therapy (SEDAR) Spanish society of emergency and emergency medicine (SEMES) and Spanish society of otolaryngology, head and neck surgery (SEORL-CCC) Guideline for difficult airway management. Part I. Rev Esp Anestesiol Reanim (Engl Ed). 2024 10.1016/j.redare.2024.02.001 38340791

[7] Gómez-Ríos M, Sastre JA, Onrubia-Fuertes X, López T, Abad-Gurumeta A, Casans-Frances R, et al. Spanish society of anesthesiology, reanimation and pain therapy (SEDAR) Spanish society of emergency and emergency medicine (SEMES) and Spanish society of otolaryngology, head and neck surgery (SEORL-CCC) Guideline for difficult airway management. Part II. Rev Esp Anestesiol Reanim (Engl Ed). 2024 10.1016/j.redare.2024.02.001 38340791

[8] Zhu Y, Zhao Y, Dou L, Guo R, Gu X, Gao R (2021). The hospital management practices in Chinese county hospitals and its association with quality of care, efficiency and finance. BMC Health Serv. Res..

[9] Ma, WH., Dai, WJ., Wang, Y. Preliminary survey on management of dificult airway in Guangdong province. J. Clin. Anesthesiol. 2014, 30(11)

[10] Ma, WH., Wang, Y., Zhong, M., Li, YH., Liu, HH. & Li, YX., et al. Investigation and analysis of difficult airways in medical institutions in China. J. Clin. Anesthesiol. 2020, 36(4)

[11] Liu HH, Wang Y, Zhong M, Li YH, Gao H, Zhang JF (2021). Managing the difficult airway: A survey of doctors with different seniority in China. Medicine (Baltimore).

[12] Apfelbaum JL, Hagberg CA, Connis RT, Abdelmalak BB, Agarkar M, Dutton RP (2022). American society of anesthesiologists practice guidelines for management of the difficult airway. Anesthesiology.

[13] Cook TM, O'Sullivan E, Kelly FE (2021). The 2004 Difficult Airway Society guidelines for the management of difficult tracheal intubation: Revolutionary and enduring. Anaesthesia.

[14] Yu BW, Wu XM, Zuo MZ, Deng XM, Gao X, Tian M (2013). Practice guidelines for management of the difficult airway. J. Clin. Anesthesiol..

[15] Zhang C, Wang S, Li H, Su F, Huang Y, Mi W (2021). Anaesthesiology in China: A cross-sectional survey of the current status of anaesthesiology departments. Lancet Reg. Health West Pac..

[16] Candlish J, Teare MD, Dimairo M, Flight L, Mandefield L, Walters SJ (2018). Appropriate statistical methods for analysing partially nested randomised controlled trials with continuous outcomes: A simulation study. BMC Med. Res. Methodol..

[17] Roth D, Pace NL, Lee A, Hovhannisyan K, Warenits AM, Arrich J (2019). Bedside tests for predicting difficult airways: An abridged Cochrane diagnostic test accuracy systematic review. Anaesthesia.

[18] Kempthorne P, Morriss WW, Mellin-Olsen J, Gore-Booth J (2017). The WFSA global anesthesia workforce survey. Anesth. Analg..

[19] Ahmad I, El-Boghdadly K, Bhagrath R, Hodzovic I, McNarry AF, Mir F (2020). Difficult airway society guidelines for awake tracheal intubation (ATI) in adults. Anaesthesia.

[20] Ma W-h (2016). The ABS algorithm and practice of dificult airway management.

[21] Fitzgerald E, Hodzovic I, Smith AF (2015). 'From darkness into light': Time to make awake intubation with videolaryngoscopy the primary technique for an anticipated difficult airway?. Anaesthesia.

[22] Goto Y, Goto T, Hagiwara Y, Tsugawa Y, Watase H, Okamoto H (2017). Techniques and outcomes of emergency airway management in Japan: An analysis of two multicentre prospective observational studies, 2010–2016. Resuscitation.

[23] Cook TM, Kelly FE (2017). A national survey of videolaryngoscopy in the United Kingdom. Br. J. Anaesth..

[24] Porhomayon J, El-Solh AA, Nader ND (2010). National survey to assess the content and availability of difficult-airway carts in critical-care units in the United States. J. Anesth..

[25] Schyma BM, Wood AE, Sothisrihari S, Swinton P (2020). Optimising remote site airway management kit dump using the SCRAM bag-a randomised controlled trial. Perioper. Med. (Lond)..

[26] Jung DTU, Grubb L, Moser CH, Nazarian JTM, Patel N, Seldon LE (2022). Implementation of an evidence-based accidental tracheostomy dislodgement bundle in a community hospital critical care unit. J. Clin. Nurs..

[27] Zhang J, Ong S, Toh H, Chew M, Ang H, Goh S (2022). Success and time to oxygen delivery for scalpel-finger-cannula and scalpel-finger-bougie front-of-neck access: A randomized crossover study with a simulated “Can't intubate, Can't Oxygenate” scenario in a manikin model with impalpable neck anatomy. Anesth. Analg..

[28] Hamaekers AE, Henderson JJ (2011). Equipment and strategies for emergency tracheal access in the adult patient. Anaesthesia.

[29] Hubble MW, Wilfong DA, Brown LH, Hertelendy A, Benner RW (2010). A meta-analysis of prehospital airway control techniques part II: Alternative airway devices and cricothyrotomy success rates. Prehosp. Emerg. Care.

[30] Kristensen MS, Teoh WH, Baker PA (2015). Percutaneous emergency airway access; prevention, preparation, technique and training. Br. J. Anaesth..

[31] McNarry AF, Cook TM, Baker PA, O’Sullivan EP (2020). The airway lead: Opportunities to improve institutional and personal preparedness for airway management. Br. J. Anaesth..

[32] Frerk C, Mitchell VS, McNarry AF, Mendonca C, Bhagrath R, Patel A (2015). Difficult airway society 2015 guidelines for management of unanticipated difficult intubation in adults. Br. J. Anaesth..

[33] Karamchandani K, Wheelwright J, Yang AL, Westphal ND, Khanna AK, Myatra SN (2021). Emergency airway management outside the operating room: Current evidence and management strategies. Anesth. Analg..

[34] Chow YM, Tan Z, Soh CR, Ong S, Zhang J, Ying H (2020). A prospective audit of airway code activations and adverse events in two tertiary hospitals. Ann. Acad. Med. Singap..

[35] Ecc Committee S, Task Forces of the American Heart A (2005). American heart association guidelines for cardiopulmonary resuscitation and emergency cardiovascular care. Circulation.

[36] Chrimes N, Higgs A, Hagberg CA, Baker PA, Cooper RM, Greif R (2022). Preventing unrecognised oesophageal intubation: A consensus guideline from the project for universal management of airways and international airway societies. Anaesthesia.

[37] Grande B, Kolbe M, Biro P (2017). Difficult airway management and training: Simulation, communication, and feedback. Curr. Opin. Anaesthesiol..

[38] Baker PA, Weller JM, Greenland KB, Riley RH, Merry AF (2011). Education in airway management. Anaesthesia.

[39] Gibbins M, Kelly FE, Cook TM (2020). Airway management equipment and practice: Time to optimise institutional, team, and personal preparedness. Br. J. Anaesth..

[40] Borg PA, Stuart C, Dercksen B, Eindhoven GB (2001). Anaesthetic management of the airway in The Netherlands: A postal survey. Eur. J. Anaesthesiol..

[41] Stringer KR, Bajenov S, Yentis SM (2002). Training in airway management. Anaesthesia.

