# Supplementary material for: National survey on the current status of airway management in China
Source: Sci Rep. 2024 Jul 7;14:15627. doi: 10.1038/s41598-024-66526-8 (PMC11228041; doi:10.1038/s41598-024-66526-8)
Supplement: Supplementary file 1 — Supplementary Information. [file 41598_2024_66526_MOESM1_ESM.pdf]

Current status of airway management in China: An update of the National Airway Survey 2022

Supplementary materials

Tables

Table S1 2022 National Airway Management Status Questionnaire and Responses

Table S2

Table S2 Comparison of difficult airway management for different levels of hospitals

Table S3 Comparison of difficult airway management for Anesthesiologists with different years of working length

Figures

Fig.S1 Bar chart of the most common airway assessment approaches used by respondents

Fig.S2 ABS difficult airway algorithm

Supplementary Tables

Supplementary Table S1 2022 National Airway Management Status Questionnaire and Responses

Questionnaire and Responses		Numbers (%)
Part 1. Basic characteristics of the respondents		
Q1. Where is your hospital from?		
	See Fig.1 for answers.	
Q2. How many years of practice do you have?		
	Less than 5 years	383(10.12%)
	5 to 10 years	683(18.05%)
	More than 10 years	2717(71.82%)
Q3. The director of the anesthesiology department		1385(36.61%)
Q4. What is the tiers of your hospital?		
	Tier 3	2546(67.30%)
	Tier 2	1157(30.58%)
	Others	80(2.11%)
Q5. Have you experienced canceling or delaying surgery due to difficult airway during the six years 2016-2022: Yes		1318(34.84%)
Q6. How can you be reached?		NA
Part 2. How to assess the airway		
Q7. What is the most common difficult airway# you encounter?		
	Difficult Facemask Ventilation	383(10.12%)
	Difficult Supraglottic Airway Ventilation	169(4.47%)
	Difficult Laryngeal Exposure	1734(45.84%)
	Difficult Tracheal Intubation	1144(30.24%)
	Difficult Tracheal Extubation	41(1.08%)
	Difficult emergency front of neck access	312(8.25%)
Q8. What are your three most common assessment approaches? 【Multiple Choice】		
	Mouth opening	3609(95.40%)
	Thyromental distance	3270(86.44%)
	Atlanto-occipital joint movement	2615(69.13%)
	Cormack–Lehane scale	1381(36.51%)
	Ultrasound	124(3.28%)
	History of difficult airway	1710(45.20%)
	Wilson risk scores	280(73.40%)
	Others	82(2.17%)
Part 3. Anticipated airway management		
Q9. What kind of management do you use for an		

anticipated difficult airway?	
AIST	2501(66.11%)
GASB	844(22.31%)
RSAI	438(11.58%)
Q10. Are anticholinergic drugs routinely used for awake tracheal intubation: Yes	2541(67.17%)
Q11. If you do not choose an awake intubation, what is the reason?	
Worried that patients will not accept	1468(38.81%)
Unfamiliarity	647(17.10%)
Long operation time	350(9.25%)
High failure rates	288(7.61%)
Patient rejection	1030(27.23%)
Q12. What is your preferred intubation device for an anticipated difficult airway?	
Flexible intubation scope (including fiberscope)	1850(48.90%)
Videolaryngoscopes	1505(39.78%)
Lightwand or optical stylets	285(7.53%)
Intubating laryngeal mask airway	129(3.41%)
Others	14(0.37%)
Q38. What do you usually do when dealing with patients with difficult airways?	
Try to intubate 1-2 times, if not, change the device and intubate again	1115(29.47%)
Try to intubate 1-2 times, if not, seek help from the superior	2074(54.82%)
Seek help right away	594(15.70%)
Q39. Do you seek help if you have a difficult airway situation?	
Routinely	2579(68.17%)
Occasionally	962(25.43%)
Handling independently	242(6.40%)
Part 4. Unanticipated airway management	
Q13. What would you prefer if the glottis could not be exposed after the use of muscle relaxants?	
Blind intubation once	865(22.87%)
Keep ventilation and wait for the superior to help	523(13.83%)
Both	2395(63.31%)
Q14. If you try to intubate once and failed, will you?	
Seek for help	1635(43.22%)
Use other devices for intubation	1805(47.71%)
Keep the patient ventilated and wait until the patient wakes up	315(8.33%)
Others	28(0.74%)

Q15. In case of difficult intubation, what is your most common ventilation device besides the facemask?	
Laryngeal mask airway	2769(73.20%)
Oropharyngeal airway	940(24.85%)
Combitube	40(1.06%)
Others	34(0.90%)
Part 5. Front of neck access emergency techniques	
Q16. Have you encountered the use of FONA technique to rescue patients during the six years 2016-2022: Yes	1161(30.69%)
Q17. When encountering an emergency where the patient Cannot intubate, Cannot ventilate (CICV), what kind of FONA would be your first choice?	
Needle cricothyrotomy	2807(74.20%)
Surgical cricothyrotomy	354(9.36%)
Tracheotomy	522(13.80%)
Others	100(2.64%)
Q18. After induction of general anesthesia, when there is a CICV situation, tracheotomy in your hospital is usually performed by?	
Anesthesiologists	457(12.08%)
Non-anesthesiologists (Surgeon)	3052(80.68%)
Others	274(7.24%)
Q19. Have you ever performed needle cricothyrotomy for the emergency airway: Yes	904(23.90%)
Q20. Have you ever performed surgical cricothyrotomy for the emergency airway: Yes	296(7.82%)
Q21. Have you ever performed tracheotomy for the emergency airway: Yes	300(7.93%)
Part 6. Difficult airway management outside the operating room	
Q22. What do you think are the top three devices you should take to intubate outside the operating room? 【Multiple Choice】	
Laryngeal mask airway	2719(71.87%)
Manual resuscitator	3094(81.79%)
Oropharyngeal airway	1982(52.39%)
Videolaryngoscope	3501(92.55%)
Flexible intubation scope	453(11.97%)
Others	55(1.45%)
Q23. Intubation outside the operating room, what's your biggest concern?	
Difficult airway	2384(63.02%)
Full Stomach	813(21.49%)
Surrounding environmental impact	172(4.55%)

Hemorrhage from the respiratory tract	405(10.71%)
Others	9(0.24%)
Q24. Outside the operating room, how do you usually identify the position of the endotracheal tube?	
Auscultation	2865(75.73%)
Chest rise	340(8.99%)
Capnography	397(10.49%)
Flexible intubation scope (including fiberscope)	162(4.28%)
Others	19(0.50%)
Part 7. Difficult airway management training	
Q25. Have you ever attended difficult airway training: Yes	2760(72.96%)
Q26. What do you think is the most important feature in difficult airway training?	
Algorithms	896(23.68%)
Actual demonstration of patients	761(20.12%)
Devices	375(9.91%)
Difficult airway case discussion	424(11.21%)
Simulated scenarios	1327(35.08%)
Q27. Have you been trained in guided retrograde intubation: Yes	1124(29.71%)
Q28. Have you been trained in needle cricothyrotomy: Yes	1964(51.92%)
Q29. Have you been trained in surgical cricothyrotomy: Yes	1049(27.73%)
Q30. Have you been trained in tracheostomy: Yes	831(21.97%)
Q31. Have you been trained in jet ventilation: Yes	1053(27.84%)
Q32. What kind of devices would you most like to be trained on?	
Laryngeal mask airway	55(1.45%)
Videolaryngoscopes	271(7.16%)
Flexible intubation scopes	1241(32.80%)
Lightwand	137(3.62%)
Emergency airway devices	2079(54.96%)
Q33. Have you read the 2022 American Society of Anesthesiologists Practice Guidelines for Management of the Difficult Airway: Yes	1979(52.31%)
Q34. How do you evaluate the American Society of Anesthesiologists' Practice Guidelines for Management of the Difficult Airway?	
Detailed and practical	1644(43.46%)
Complex and difficult to remember	1669(44.12%)
Others	470(12.42%)
Q35. Which of the following difficult airway	

management algorithm are you most familiar with?	
American Society of Anesthesiologists (ASA) difficult airway management algorithm	174(4.60%)
Chinese Society of Anesthesiologists (CSA) difficult airway management algorithm	1037(27.41%)
ABS algorithm for difficult airway management	2572(67.99%)
Q36. What do you think is the biggest feature of the ABS algorithm for difficult airway management?	
Practical	2419(63.94%)
Safety	608(16.07%)
Easy to remember	721(19.06%)
Others	35(0.93%)
Q37. Has your department ever organized a group study on any guidelines for airway management: Yes	1116(80.58%)
Q38. Does your department regularly (at least once a year) arrange for anesthesiologists in your department to attend airway training: Yes	672(48.52%)
Q39. Does your department have a regular (at least quarterly) practice of sharing difficult airway cases within the department: Yes	715(51.62%)
Part 8. Availability of airway devices and emergency airway devices*	
Q40. Are you the director of the Anesthesiology Department: Yes	1386(36.61%)
Q41. Does your department have videolaryngoscopes: Yes	1346(97.18%)
Q42. Does your department have lightwands or optical stylets: Yes	834(60.22%)
Q43. Does your department have flexible intubation scope (including fiberscope): Yes	871(62.89%)
Q44. Does your department have bougies or airway exchange catheters: Yes	472(34.08%)
Q45. Does your department have laryngeal masks: Yes	1329(95.96%)
Q46. Does your department have video laryngeal masks: Yes	195(14.08%)
Q47. Does your department have video endotracheal tubes: Yes	166(11.99%)
Q48. Does your department have jet ventilation devices: Yes	200(14.44%)
Q49. Does your department have the needle cricothyrotomy kit: Yes	501(36.17%)
Q50. Does your department have the surgical cricothyrotomy kit: Yes	429(30.97%)
Q51. Does your department have the difficult airway management emergency kit or cart: Yes	676(48.81%)

*Part 9. Adverse events related to difficult airway**	
Q52. Will you inform family members or patients in writing or verbally after surgery if there is a difficult airway?	
Written notification	293(7.75%)
Oral notification	1273(33.65%)
Both	2054(54.30%)
No notification	163(4.31%)
Q54. In the past 6 years (2016-11~2022-11), has your department ever found the following problems due to difficult airway? †	
Cardiac arrest but without sequelae	88(6.35%)
Brain damage	40(2.89%)
Death	31(2.24%)
None of the above occurred	31(2.24%)

* These questions can only be answered by the director of the Department of Anesthesiology.

# The definition of a difficult airway is as follows[1, 2].

Difficult Facemask Ventilation: Inability to provide adequate ventilation (absence of end-tidal carbon dioxide detection waveform) to the patient due to one or more of the following reasons: insufficient mask seal, excessive gas leakage, or excessive resistance to gas flow entry or exit. Signs of inadequate ventilation include (but are not limited to): no or inadequate chest movement, absent or inadequate breath sounds on auscultation, signs of severe obstruction, cyanosis, gastric air entry or dilatation, decreasing or inadequate oxygen saturation, absent or inadequate exhaled gas flow as measured by spirometry, anatomic lung abnormalities as detected by lung ultrasound, and hemodynamic changes associated with hypoxemia or hypercarbia (e.g., hypertension, tachycardia, bradycardia, arrhythmia).

Difficult Supraglottic Airway Ventilation: Adequate ventilation cannot be provided because of one or more of the following issues: difficult supraglottic airway placement, the need for multiple attempts to place the supraglottic airway, inadequate sealing of the supraglottic airway, excessive gas leakage, or excessive resistance to gas entry or exit.

Difficult Laryngeal Exposure: After several attempts at direct laryngoscopy or videolaryngoscopy, it is impossible to see any portion of the vocal cords.

Difficult Tracheal Intubation: Tracheal intubation requires multiple attempts, additional operators, devices, and/or assistive techniques to be successful or failure of tracheal intubation after multiple attempts.

Difficult Tracheal Extubation: The loss of airway patency and adequate ventilation after removal of a tracheal tube or supraglottic airway from a patient with a known or suspected difficult airway (i.e., an “at risk” extubation).

Difficult emergency front of neck access: Anatomic features or abnormalities reduce or prevent the likelihood of successfully placing an airway into the trachea through the front of the neck.

† Our survey was conducted on a per-hospital basis, not general anesthesia cases. According to the China Health Statistics Yearbook 2022 (http://www.nhc.gov.cn), the number of medical institutions covered by our survey is about 75,477. It showed an event rate of 5.13%

for brain damage or death. The number of general anesthesia surgeries in China in 2022 is about 70 million, so the number of brain damage or death is roughly 3,840 (5.13%*75,477), which translates into a ratio of 1 in 180,000 general anesthesia cases (3,840 cases/70million general anesthesia cases), which approximates the findings of NAP4[3].

FONA: emergency Front of neck access; AIST: Awake intubation under sedation and topical anesthesia; GASB: General anesthesia with preserved spontaneous breathing; RSAI: rapid sequence anesthesia induction.

Supplementary Table S2 Comparison of difficult airway management for different levels of hospitals

	Tier 3 hospitals (N=2546)	Other hospitals (N=1237)	*P*-value
How to manage an anticipated difficult airway			
AIST	1812(71.17%)	689(55.70%)	<0.001
GASB	531(20.86%)	313(25.30%)	0.002
RSAI	203(7.97%)	235(19.00%)	<0.001
What is the preferred intubation device in the anticipated difficult airway			
Flexible intubation scopes	1423(55.89%)	427(34.52%)	<0.001
Video laryngoscopes	896(35.19%)	609(49.23%)	<0.001
Lightwand or optical stylets	159(6.25%)	126(10.19%)	<0.001
Intubating laryngeal mask	62(2.44%)	67(5.42%)	<0.001
Others	6(0.24%)	8(0.64%)	0.082
Have you ever attended difficult airway training: Yes	1912(75.09%)	848(68.55%)	<0.001
Have you ever received the below training			
Retrograde intubation: Yes	788(30.95%)	336(27.16%)	0.018
Needle cricothyrotomy: Yes	1408(55.30%)	556(44.95%)	0.028
Surgical cricothyrotomy: Yes	735(28.87%)	314(25.38%)	0.852
Tracheotomy: Yes	562(22.07%)	269(21.75%)	0.001
Jet ventilation: Yes	788(30.95%)	255(21.42%)	<0.001

AIST: Awake intubation under sedation and topical anesthesia; GASB: General anesthesia with preserved spontaneous breathing; RSAI: rapid sequence anesthesia induction.

Supplementary Table S3 Comparison of difficult airway management for Anesthesiologists with different years of working length

	AM (N=2717)	AL (N=1066)	*P*-value
How to manage an anticipated difficult airway			
AIST	1820(66.99%)	681(63.88%)	0.086
GASB	613(22.56%)	231(21.67%)	0.598
RSAI	284(10.45%)	154(14.45%)	0.001
What is the preferred intubation device in the anticipated difficult airway			
Flexible intubation scopes	1242(45.71%)	608(57.04%)	<0.001
Videolaryngoscopes	1137(41.85%)	368(34.52%)	<0.001
Lightwand or optical stylets	221(8.13%)	64(6.00%)	0.030
Intubating laryngeal mask	106(3.90%)	23(2.16%)	0.010
Others	11(0.41%)	3(0.28%)	0.769
Have you ever attended difficult airway training: Yes	2076(76.41%)	684(64.17%)	<0.001
Have you ever performed below FONA technique			
Needle cricothyrotomy: Yes	764(28.12%)	140(13.13%)	<0.001
Surgical cricothyrotomy: Yes	233(8.58%)	63(5.91%)	0.007
Tracheotomy: Yes	224(8.24%)	76(7.13%)	0.283

AIST: Awake intubation under sedation and topical anesthesia; GASB: General anesthesia with preserved spontaneous breathing; RSAI: Rapid sequence anesthesia induction; FONA: Front of neck access.

Supplementary figures

Supplementary Fig.S1 Bar chart of the most common airway assessment approaches used by respondents

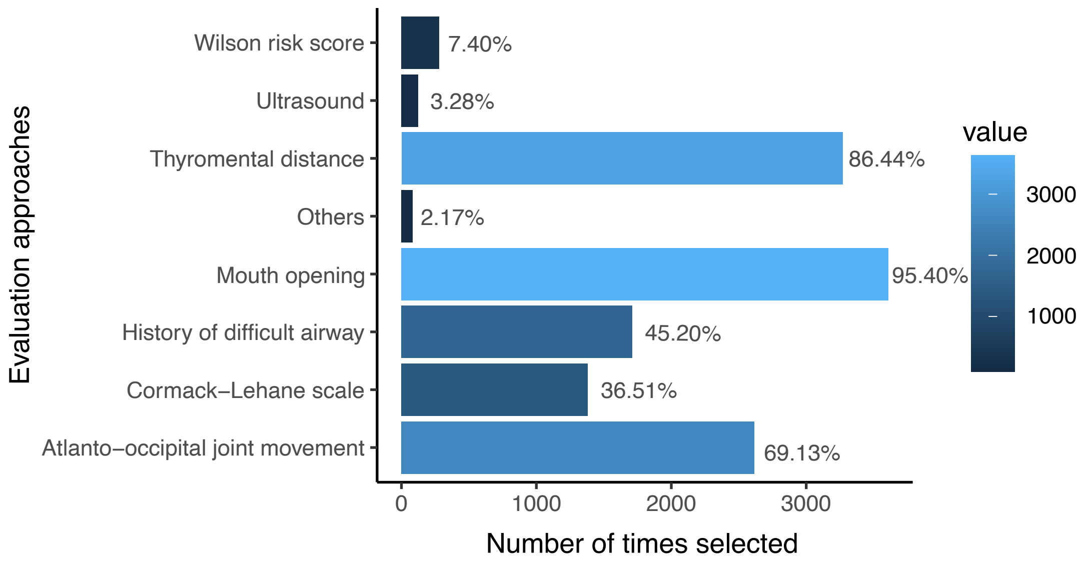

Supplementary Fig.S2 ABS difficult airway algorithm

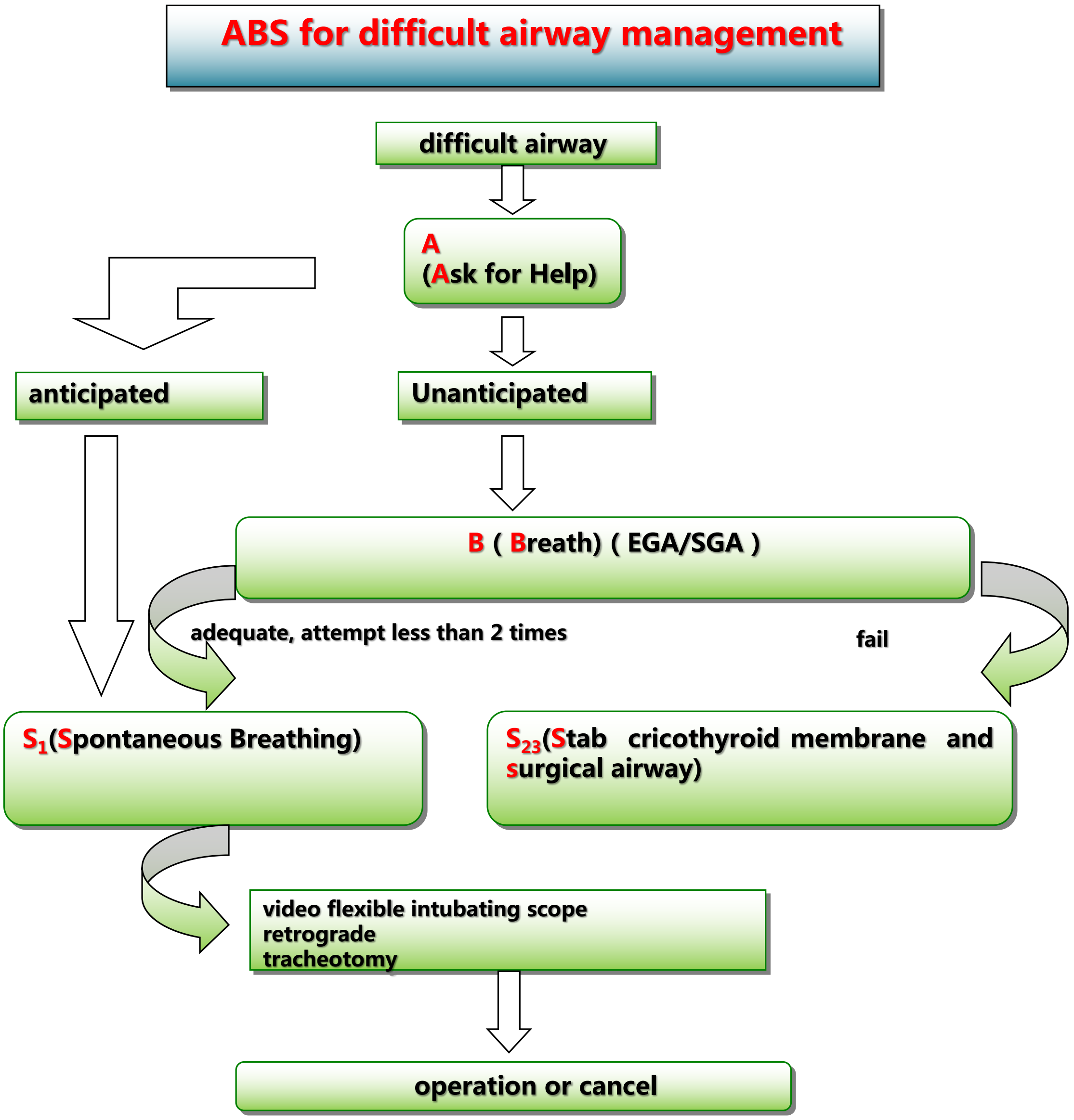

References

1. Apfelbaum JL, Hagberg CA, Connis RT, Abdelmalak BB, Agarkar M, Dutton RP, et al. 2022 American Society of Anesthesiologists Practice Guidelines for Management of the Difficult Airway. Anesthesiology. 2022;136(1):31-81.
2. Gómez-Ríos M, Sastre JA, Onrubia-Fuertes X, López T, Abad-Gurumeta A, Casans-Frances R, et al. Spanish Society of Anesthesiology, Reanimation and Pain Therapy (SEDAR) Spanish Society of Emergency and Emergency Medicine (SEMES) and Spanish Society of Otolaryngology, Head and Neck Surgery (SEORL-CCC) Guideline for difficult airway management. Part I. Rev Esp Anestesiol Reanim (Engl Ed). 2024.
3. Cook TM, Woodall N, Harper J, Benger J. Major complications of airway management in the UK: results of the Fourth National Audit Project of the Royal College of Anaesthetists and the Difficult Airway Society. Part 2: intensive care and emergency departments. Br J Anaesth. 2011;106(5):632-42.
